# Antiaging and Anxiolytic Effects of Combinatory Formulas Based on Four Medicinal Herbs

**DOI:** 10.1155/2017/4624069

**Published:** 2017-03-28

**Authors:** Rui Li, Wingman Chan, Waikin Mat, Yiucheong Ho, Rigil K. Yeung, Shuiying Tsang, Hong Xue

**Affiliations:** ^1^Division of Life Science, Hong Kong University of Science and Technology, Clear Water Bay, Hong Kong; ^2^Applied Genomics Center, Hong Kong University of Science and Technology, Clear Water Bay, Hong Kong

## Abstract

The objective of the present study was to search for medicinal-herb combinations based on Radix* Bupleurum chinense* DC (“B”), Rhizoma* Corydalis yanhusuo* WT Wang (“Y”), Caulis* Polygonum multiflorum* Thunb (“P”), and Flos* Albizia julibrissin* Durazz (“A”) for antiaging, anxiolytic, and sedative effects. Application of the D-galactose induced accelerated-aging model employing male ICR mice showed that oral administration of some combinations of B, Y, P, and A significantly improved spatial memory in Y-maze test and reduced brain levels of tumor necrosis factor-*α* and interleukin-6 based on immunoassays and oxidative stress marker malondialdehyde, based on the thiobarbituric acid test, and the loss of whiskers, indicating antiaging and antineurodegeneration effects. In addition, some of the combinatory formulas induced anxiolysis measured using the elevated plus-maze test and/or sedative effects measured using the hole-board test. Over the range of dosages examined, all possible combinations of the four herbs were devoid of any significant side effects in the form of altered locomotor activity, decreased muscle coordination, or anterograde amnesia assessed using the photobeam and rotarod and step-through passive avoidance methods, respectively. The results suggest that various combinations of the B, Y, P, and A herbs could be useful as nonsedative, antiaging and/or antineurodegenerative agents, or anxiolytic agents.

## 1. Introduction

The world's population is aging rapidly, with the over-65 age group outnumbering the below-15 age group in a fast increasing number of countries. Age-related cognitive decline and neurodegeneration are therefore becoming foremost health and social concerns. Physiologically, damage to the structures of the medial temporal lobe including the hippocampus contributes to a deficit in spatial memory [1, 2], and oxidative damage by reactive oxygen species (ROS) is commonly associated with aging [3, 4]. As well, proinflammatory agents such as interleukin-6 (IL-6), C-reactive protein, and tumor necrosis factor-alpha (TNF-*α*) can enhance resistance to infectious disease but also susceptibility in old age to a chronic low-grade inflammation, or “inflammaging,” that constitutes a highly significant factor for both morbidity and mortality [5, 6]. Long-lived people, especially centenarians, seem to cope with inflammaging through an “anti-inflammaging” cytokine response that may represent a key to longevity [7]. Therefore, treatments that can alleviate spatial memory deficit, relieve oxidative stress, and lower the levels of proinflammatory agents such as TNF-*α* and IL-6 could be important for reducing some of the effects of aging. Since old people are often beset as well by such neurophysiological conditions as anxiety and sleeplessness, which are aggravated by the feelings of helplessness and loneliness common to old age, treatments for anxiety and sleep disorders are equally fundamental for the health of the aged.

The health problems arising from aging, age-related neurodegenerative diseases, anxiety disorders, and sleep disorders require therapeutic agents that can cross the blood-brain barrier and are relatively free of adverse side effects so that they can be administered on a chronic basis. Traditional Chinese Medicine (TCM) employs approximately 500 medicinal herbs, and multiherb combinations comprising complementary herbs are usually favored over single herbs [8–11]. The toxicities of these herbs are also either limited or at least well understood from centuries of usage. Previously, our search for an anxiolytic herbal formula has led to the development of the “BYPA,” or Erhuhuanteng, combination comprising the four Chinese medicinal herbs Radix* Bupleurum chinense* DC (“B”), Rhizoma* Corydalis yanhusuo* WT Wang (“Y”), Caulis* Polygonum multiflorum* Thunb (“P”), and Flos* Albizia julibrissin* Durazz (“A”) [12]. The B, Y, P, and A herbs are widely employed in TCM. The contents of B include saikosaponin A which displays anti-inflammatory, anticancer, and antioxidative effects [13–15], and saikosaponin D which induces immunomodulatory and anticancer effects [16, 17]. Y contains dl-tetrahydropalmatine which induces anxiolytic effects in mice when administered orally [18]. The l-tetrahydropalmatine isomer is a dopamine receptor antagonist with potential application to treatment of drug addiction [19–22] and amelioration of anxiety and depression-related symptoms of posttraumatic stress disorder in rats [23]. P is employed as a sedative for neurasthenia and insomnia and to activate blood circulation in collaterals for treatment of aching limbs. It can induce hypnotic effects when coadministered with pentobarbital [24]. A is employed as sedative and tranquilizer. It increases pentobarbital-induced sleeping time in a dose dependent manner [25, 26] and exhibits antidepressant effect in the forced swim test [27]. The anxiolytic effect of the BYPA formula also clearly indicates that its four constituent herbs contain neuroactive ingredients that can cross the blood-brain barrier. Accordingly, herbal combinations based on B, Y, P, and A have been examined in the present study regarding their potential utility for treating the effects of normal aging, age-related degenerative diseases, anxiety disorders, and sleep disorders.

To examine the effects of herbal formulas comprising combinations of B, Y, P, and A on aging-related changes, the d-galactose-induced aging model was employed, which is based on the findings that chronic exposure to d-galactose not only causes shortened lifespan in Drosophila and housefly [28, 29], but also brings about neurodegeneration, increased oxidative stress in the brain, and spatial memory impairment [30, 31]. The application of this accelerated-aging model has led to such findings as the amelioration of cognitive dysfunction and neurodegeneration by R-*α*-lipoic acid [30], prevention of cognitive impairment and hippocampus senescence by ginsenoside Rg1 [32], and reduction of elevated brain ROS, TNF-*α*, and IL-6 by asiatic acid [33]. Analysis of anxiety and sedation was carried out using the elevated plus-maze test and hole-board test, respectively.

## 2. Materials and Methods

### 2.1. Chemical Compounds

Radioactive [^3^H]-flunitrazepam (Methyl-[^3^H], 81.4 Ci/mmol), [^3^H]-muscimol (Methylene-[^3^H], 25.5 Ci/mmol), and [^3^H]-spiperone (Benzene ring-[^3^H], 15.7 Ci/mmol) were obtained from Perkin-Elmer (Life Sciences, Inc., Boston, MA). Dilutions were made using 50 mM Tris-Cl, pH 7.4, and they were stored at 4°C. Diazepam (DZ) and GABA were obtained from Sigma (St. Louis, MO, USA). Diazepam was dissolved in 0.9% NaCl in the presence of 1% DMSO for animal tests. These drugs were administered to mice in a delivery volume of 10 ml/kg through the oral route. d-galactose, malondialdehyde (MDA), and thiobarbituric acid (TBA) were obtained from Sigma (St. Louis, MO, USA), and d-galactose was dissolved in 0.9% NaCl for animal treatment.

### 2.2. Animals

Male ICR mice from the Animal and Plant Care Facility, Hong Kong University of Science & Technology (HKUST), were housed in groups of five to ten with food and water ad libitum and kept on 08:00- to 20:00-light/dark cycle. In the experiments, the mice were separated randomly into different groups; and the number of mice used in each group in the different experiments is described in Sections 2.4, 2.6, 2.7, 2.10, and 2.11. All animal experiments were preapproved by the HKUST Animal Ethics Committee and conducted in accordance with the Code of Practice for Care and Use of Animals for Experimental Purposes approved by the Animal Welfare Advisory Group, the Agriculture, Fisheries, and Conservation Department, and the Department of Health of Hong Kong Special Administrative Region (Animal ethical code number: HKUST #2015029).

### 2.3. Preparation of Herbal Extracts

To prepare the four-herb BYPA extract, 65 g B, 45 g Y, 45 g P, and 45 g A were powdered and boiled in 2 L of 5% acetic acid for 2 hours. The supernatant was collected and the residue was reboiled in 2 L of 5% acetic acid for 2 hours. The two batches of supernatant were pooled together, filtered, and boiled down to a volume of approximately 100 ml prior to drying in oven at 92°C. Thereupon 300 ml 100% ethanol was added to the finely powdered dried residue and redried at 92°C. The dried extract was weighed and powdered.

To prepare an extract from a subset of the four constituent herbs, one or more of the herbs were omitted in performing the same procedure; for example, only 65 g B, 45 g Y, and 45 g P were boiled in 2 L of 5% acetic acid for 2 hours and so on in preparing the three-herb BYP extract, or only 45 g P and 45 g A were boiled in 2 L of 5% acetic acid for 2 hours and so on in preparing the two-herb PA extract.

### 2.4. d-Galactose-Induced Accelerated-Aging Model

10-week-old ICR male mice were randomly separated into groups, vehicle group (Veh, *n* = 20), d-galactose treated control group (d-gal, *n* = 20), and herbal extract-treated groups (*n* = 12/group). Mice were given daily 150 mg/kg d-galactose by subcutaneous injection, accompanied by either daily oral administration of the vehicle (0.9% NaCl) in the d-gal group or daily oral administration of one of the BYPA, BYP, BYA, BPA, YPA, BY, BP, and YP herbal extracts (120 mg/kg), for an exposure period of 8 weeks. The Veh group received daily injection of saline (0.9% NaCl) and also daily oral administration of saline (0.9% NaCl) for 8 weeks.

### 2.5. Y-Maze Test

**Figure 1:**
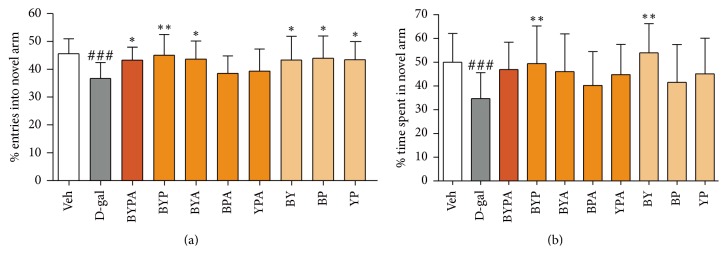
Spatial memory impairment in d-galactose-induced aging mice. d-gal mice were given daily 150 mg/kg d-galactose by subcutaneous injection with or without daily oral coadministration of different combinatorial herbal formulas (120 mg/kg) for eight weeks prior to the Y-maze test. Decreased percentile entry into novel arm (a) or time spent in novel arm (b) compared with vehicle-treated group (Veh) represented spatial memory impairment. Data are expressed in mean ± SD. ^###^(*p* < 0.001) indicates significant difference between d-gal group and Veh group, whereas ^*∗*^(*p* < 0.05) or ^*∗∗*^(*p* < 0.01) indicates significant difference between herbal group and d-gal group, based on one-way ANOVA test followed by Newman-Keuls test.

The test, a two-trial memory task based on a free choice exploration paradigm in a Y-maze, avoids the use of electric shock or deprivation and the need for learning of any rule. Hippocampal damage and chronic stress have been shown to cause impaired spatial memory performance in the Y-maze [34, 35]. In the study illustrated in Figure 1, the test was given to each animal at the end of the 8-week exposure to d-galactose using a Y-maze that consisted of three identical arms spaced at an angle of 120° to one another, designated, respectively, as the “start arm,” “open arm,” and “novel arm.” At the start of the test, the entrance to the novel arm was closed off, and animal was placed into the start arm and allowed to explore the start arm and the open arm freely for 10 minutes. The animal was removed from the maze for 60 minutes before being reintroduced into the maze, now with the entrance to the novel arm also opened up so that the animal could freely explore all three arms for a test period of 5 minutes. During this test period, the number of entries made by the animal into each of the three arms was counted, and the total time spent by the animal in each of the three arms was recorded, where any arm entry or arm exit was defined by the placement of all four paws inside or outside the arm. Animals with total loss of spatial memory would enter into the three arms randomly, making ~33% of arm entries into the novel arm and spending ~33% of the time in the novel arm. In contrast, animals with either no loss or only partial loss of spatial memory would enter into and also spend time in the novel arm preferentially as unexplored territory, causing both of these percentages to exceed 33%.

### 2.6. Neuroinflammation and Oxidative Stress in Brain

Mice were sacrificed by euthanasia at the end of the 8-week d-galactose exposure period (*n* = 6/group), and the brain was removed and homogenized in phosphate buffer saline, pH 7.2. The levels of TNF-*α* and IL-6 in the brain were measured as neuroinflammation markers using ELISA with solid phase sandwich kits (Invitrogen Corporation, Camerillo, CA, USA). The minimum detectable levels were 3 pg/ml for each of TNF-*α* and IL-6. To assess oxidative stress, the level of the lipid peroxidation product MDA, a widely employed marker for oxidative stress in animal tissues, was estimated using the spectrophotometric assay at 532 nm for reaction between TBA and MDA [36, 37].

### 2.7. Elevated Plus-Maze Test

4–6-week-old male ICR mice were randomly separated into groups. Vehicle (Veh, namely, 0.9% NaCl, *n* = 20) 30, 60, 90, or 120 mg/kg herbal extract (*n* = 20) or 1 or 3 mg/kg DZ (*n* = 15) was orally administered 35 minutes prior to experiment. The test apparatus consisted of four arms, 25 × 5 cm each, extending from a central 5 × 5 cm platform in the shape of a plus sign. Two opposing arms were enclosed by 20 cm opaque high walls, making up the closed arms. The plus-maze was elevated 40 cm above ground. Each mouse was placed on to the center of the maze with head facing an open arm. The frequency and time spent in the open and closed arms were recorded for a period of 5 minutes. An arm entry was recorded when all four paws were inside the arm. At the end of each test, the apparatus was thoroughly cleansed and dried before the start of the next test. An increased percentage of time spent in open arms compared with vehicle-treated controls signified an anxiolytic effect [38, 39].

To test whether the anxiolytic effect elicited by herbal extract could be blocked by the BZ-site antagonist flumazenil, 4–6-week-old male mice were randomly separated into three groups. At 35 minutes prior to elevated plus-maze test, the first group (*n* = 25) received vehicle (0.9% NaCl, po), whereas the second group (*n* = 18) and the third group (*n* = 20) received herbal extract (po). At 15 minutes prior to elevated plus-maze test, the first and second groups received vehicle (ip) whereas the third group received 1.25 mg/kg flumazenil (i.p).

### 2.8. Hole-Board Test

After the elevated plus-maze test, the test mice were subjected to the hole-board test. The hole-board apparatus consisted of a wooden box, 60 × 60 × 20 cm, with four holes of 3 cm diameter evenly spaced on the floor. Each mouse was placed at the center of the hole-board, and the number of head-dips into the holes was recorded for a period of 5 minutes. A head-dip was scored when the head of the mouse was dipped into the hole to the extent that the eyes of the mouse passed below the hole on the floor. After each trial, the floor of the apparatus was thoroughly cleansed and dried before the start of the next test. Decreased number of head-dips compared with Veh controls was indicative of a sedative effect [40].

### 2.9. Locomotor Activity Test

Following the elevated plus-maze and hole-board tests, the test mice were further assessed for locomotor activity by means of the ZIL-2 apparatus (Beijing Institute of Materia Medica). The test apparatus, with dimensions of 60 cm × 60 cm × 12 cm, consisted of a transparent plastic cylindrical container equipped with three evenly spaced infrared beams with photodetectors. The number of transitions across the photocell beams was recorded automatically over a period of 5 minutes. An increase or decrease in the number of transitions indicated an increase or decrease in locomotor activity, respectively [41].

### 2.10. Rotarod Test

4–6-week-old male ICR mice were randomly separated into groups. Vehicle (Veh, namely, 0.9% NaCl, *n* = 20) 120 mg/kg herbal extract (*n* = 15) or 1 or 3 mg/kg DZ (*n* = 15) was orally administered 35 minutes prior to the test. The rotarod test for motor coordination utilized a custom-built apparatus consisting of a cylindrical rotarod (2.5 cm diameter) with a textured surface placed 0.5 m above the ground. Prior to vehicle or extract administration, the mice were first trained to stay for 2 min on the rotarod revolving at 16 rpm. To test the effect of vehicle or extract treatment, the time each treated mouse remained on the rotarod was recorded, with cut-off set at 2 min such that any mouse that remained on the rotarod after 2 min was accorded a time on rotarod of 2 min [42].

### 2.11. Step-through Passive Avoidance Test

4–6-week-old male ICR mice were randomly separated into groups. Vehicle (Veh, namely, 0.9% NaCl, *n* = 20), 120 mg/kg herbal extract (*n* = 15), or 1 or 3 mg/kg DZ (*n* = 15) was orally administered 35 minutes prior to training. The apparatus was a two-chamber box (Chinese Academy of Chinese Medical Science, Beijing) with one of the chambers being opaque (darkened) and the other transparent (lighted). In the training trials, each mouse was placed into the lighted chamber, and the door connecting the lighted and darkened chambers was opened 10 seconds later. Mice that did not enter the darkened chamber within 15 seconds were excluded from the experiment. After the mouse entered the darkened chamber, the door was closed and a 2-second electric foot shock of 0.4 mA was delivered through the grid floor. Ten seconds later the mouse was transferred from the darkened chamber back to the home cage. After an interval of 24 hours, it was returned to the lighted chamber. The door to the darkened chamber was opened 10 seconds later, and the time taken for the mouse to enter the darkened chamber was recorded as the step-through latency; cut-off was set at 300 seconds, such that the step-through latency for any mouse that did not enter the darkened chamber by 300 seconds was recorded as 300 seconds. Significant decrease in step-through latency indicated anterograde amnesia [43].

### 2.12. Preparation of Synaptosomal Membrane

Sprague Dawley rats (~200 g) were decapitated and the whole brains were rapidly removed and frozen in liquid nitrogen. The tissue was thawed in 20 volumes of ice-cold 0.32 M sucrose and homogenized in an ULTRA-TURRAX homogenizer. The homogenate was centrifuged at 1000 ×g for 10 min at 4°C, and the supernatant was again centrifuged at 20,000 ×g for 20 minutes at 4°C. The pellet from the second centrifugation was resuspended in 50 mM Tris-Cl, pH 7.4, to a concentration of 1 mg/ml and stored at −80°C until use. For the [^3^H]-muscimol and [^3^H]-spiperone binding assays, the pellet from the second centrifugation was further homogenized, stirred on ice with 20 volumes of ice-cold double-distilled water, and centrifuged at 48,000 ×g for 20 minutes at 4°C; this was repeated twice before resuspending the final pellet in 50 mM Tris-Cl, pH 7.4, to a concentration of 1 mg/ml.

### 2.13. Radioligand Binding Assays

In these assays, 1 nM [^3^H]-flunitrazepam, 3 nM [^3^H]-muscimol, or 1 nM [^3^H]-spiperone was incubated with 0.2 mg/ml synaptosomal membranes and different concentrations of herbal extract in 50 mM Tris-Cl, pH 7.4 at either 4°C for 90 min for [^3^H]-flunitrazepam or [^3^H]-muscimol or 25°C for 60 min for [^3^H]-spiperone binding. Subsequently the incubation mixture was placed on a Whatman GF/B filter on a Brandel 24-well harvester and washed with ice-cold 50 mM Tris-Cl, pH 7.4. Each washed filter was incubated with 4 ml scintillation cocktail for at least 60 minutes before measurement of radioactivity in a Beckman-Coulter LS6500 scintillation counter. Half-maximal inhibition concentrations (IC_50_) were determined by nonlinear regression analysis performed using Prism 5.0 (Graphpad Software). To assess nonspecific binding, 1 nM [^3^H]-flunitrazepam, 3 nM [^3^H]-muscimol, or 1 nM [^3^H]-spiperone was incubated with synaptosomal membranes and 10 *μ*M diazepam, 10 *μ*M GABA, or 10 *μ*M (+)-butaclamol, respectively, in 50 mM Tris-Cl, pH 7.4, at either 4°C for 90 min for [^3^H]-flunitrazepam or [^3^H]-muscimol or 25°C for 60 min for [^3^H]-spiperone; under these conditions, the [^3^H]-ligand bound in each instance represented nonspecific binding.

### 2.14. Statistical Analysis

Results were expressed as mean ± standard deviation (SD). One-way ANOVA test followed by Newman-Keuls test was performed on behavioral data. Binding assay data were expressed as mean ± SD of four independent incubations, each in duplicate samplings. Binding affinity was determined by nonlinear regression analysis. All statistical analyses were performed using Prism 5.0 (Graphpad Software).

## 3. Results

### 3.1. Antiaging Effects in d-Galactose-Induced Mouse Aging Model

In this model, over an 8-week d-galactose exposure period, the vehicle group (“Veh”) of animals received daily injection and oral administration of vehicle (0.9% NaCl); the d-galactose group (“d-gal”) received daily injection of d-galactose (150 mg/kg) and oral administration of vehicle; and each herbal group received daily injection of d-galactose plus oral administration of herbal extract prepared from two or more of the B, Y, P, and A herbs (see Section 2.4).

In the Y-maze test, the d-gal animals displayed a significant decrease in entries into and time spent in the novel arm of the maze with *p* < 0.001, indicating a loss of spatial memory compared with the Veh group (Figures 1(a) and 1(b)). Daily oral treatment with a BYPA, BYA, BY, BP, or YP extract accompanying d-galactose injection increased the percentile novel arm entries compared with that of the d-gal mice to *p* < 0.05; and oral treatment with BYP increased the percentile novel arm entries compared with that of the d-gal mice to *p* < 0.01. Daily oral treatment with BYP or BY also increased the percentile time spent in the novel arm compared with the d-gal mice to *p* < 0.01.

**Figure 2:**
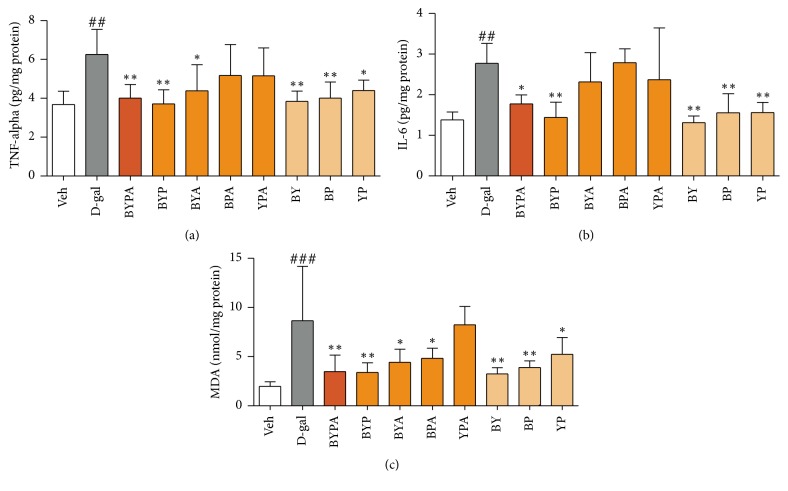
Anti-inflammatory and antioxidative effects in brains of d-galactose-induced aging mice. Different groups of mice were treated as described in Figure 1. The anti-inflammatory effect was evaluated by measurement of the brain levels of TNF-*α* (a) and IL-6 (b), and antioxidant effect was evaluated by measurement of the brain level of MDA (c). Data are expressed in mean ± SD. ^##^(*p* < 0.01) or ^###^(*p* < 0.001) indicates significant difference between d-gal group and Veh group, whereas ^*∗*^(*p* < 0.05) or ^*∗∗*^(*p* < 0.01) indicates significant difference between herbal group and d-gal group, based on one-way ANOVA test followed by Newman-Keuls test.

The levels of TNF-*α* and IL-6 were increased in the brains of d-gal mice compared with Veh mice (*p* < 0.01). Daily oral cotreatment with BYA or YP decreased the brain level of TNF-*α* compared with that of the d-gal mice to *p* < 0.05; and cotreatment with BYPA, BYP, BY, or BP decreased the brain level of TNF-*α* compared with that of the d-gal mice more extensively to *p* < 0.01 (Figure 2(a)). Daily oral cotreatment with BYPA decreased the brain level of IL-6 compared with that of the d-gal mice to *p* < 0.05; and cotreatment with BYP, BY, BP, or YP decreased the brain level of IL-6 compared with that of the d-gal mice more extensively to *p* < 0.01 (Figure 2(b)).

When the level of oxidative stress in the brain was assessed, the marker compound MDA was found to be increased in the brains of d-gal mice compared with Veh mice (*p* < 0.001). Daily oral cotreatment with BYA, BPA, or YP effectively decreased the brain MDA compared with that of the d-gal mice to *p* < 0.05; and cotreatment with BYPA, BYP, BY, or BP decreased the brain level of MDA compared with that of the d-gal mice more markedly to *p* < 0.01 (Figure 2(c)).

**Figure 3:**
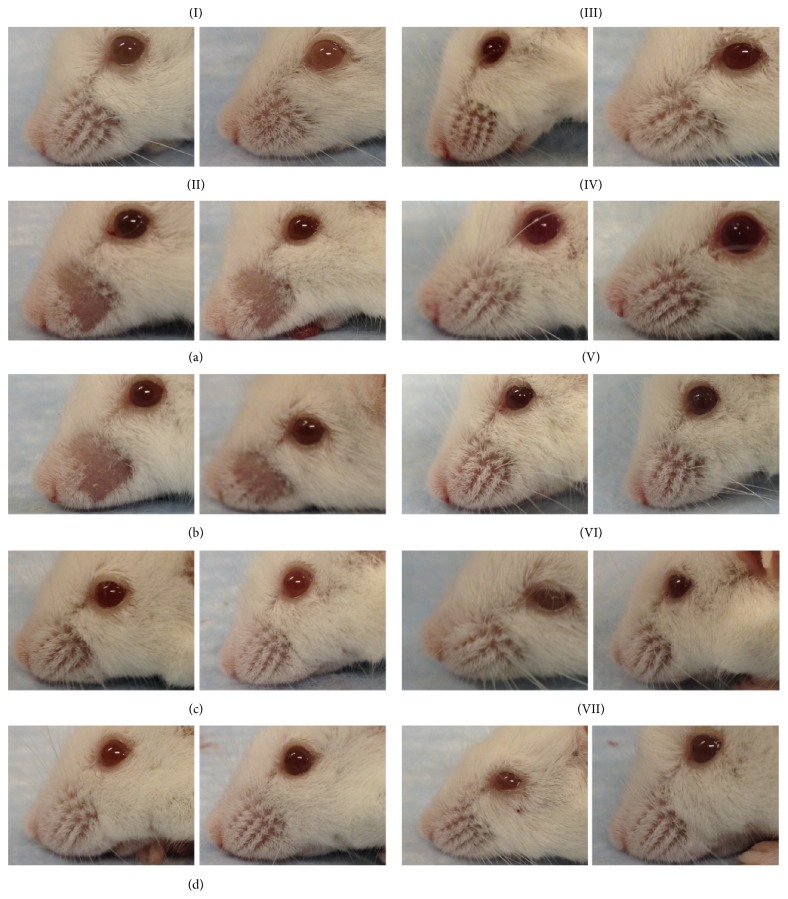
Loss of whiskers by d-galactose-induced aging mice. (I) Photographs of two Veh mice: (IIa, IIb) four d-galactose treated mice that showed loss of whiskers and (IIc, IId) four d-galactose treated mice that did not show loss of whiskers. (III) Two of the mice treated with d-galactose and BYPA, (IV) two of the mice treated with d-galactose and BYP, (V) two of the mice treated with d-galactose and BYA, (VI) two of the mice treated with d-galactose and BPA, and (VII) two of the mice treated with d-galactose and YPA.

Mice treated with d-galactose for 8 weeks showed variable extents of loss of whiskers.  Table 1 shows one particularly sensitive batch of mice where loss of whiskers was displayed by as many as 50% of the d-gal mice. In contrast, none of the mice in the Veh group, or in the groups that received d-galactose injections along with an extract from BYPA, BYP, BYA, BPA, or YPA, displayed any loss of whiskers. Thus exposure of sensitive mice to d-galactose inflicted damage to facial tissues resulting in loss of whiskers, in accord with an earlier report of coarse fur and severe hair loss in d-galactose treated mice [44]. On this basis, the findings in Table 1, also illustrated in Figure 3, suggest that the protective antiaging effects of the BYPA, BYP, BYA, BPA, and YPA extracts were not confined to brain tissues but extended significantly to facial skin as well (*p* = 0.014).

### 3.2. Anxiolytic and Sedative Effects

**Figure 4:**
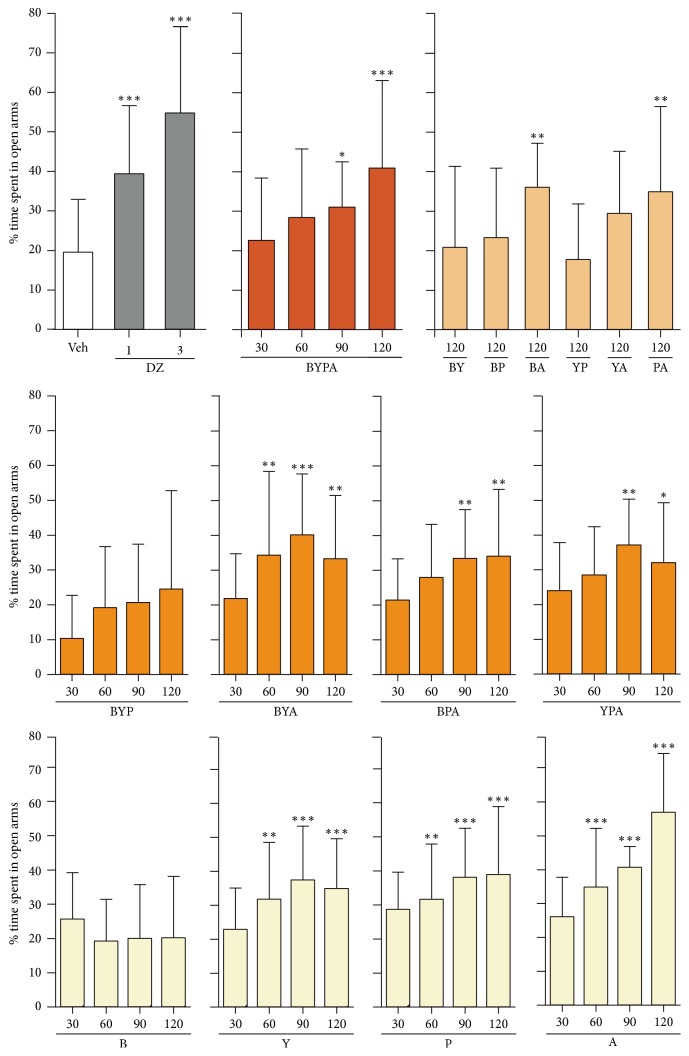
Anxiolytic effect of different combinations of B, Y, P, and A herbs. The performances of mice treated with saline vehicle (Veh), diazepam (DZ), and different single-herb or multiherb extracts were assessed with respect to anxiolytic effect using the elevated plus-maze test, where increased percentage time spent in open arms compared with Veh represented an anxiolytic effect. Data are expressed in mean ± SD of the percentage of time spent in open arms. ^*∗*^(*p* < 0.05), ^*∗∗*^(*p* < 0.01), or ^*∗∗∗*^(*p* < 0.001) indicates significant difference from Veh group based on one-way ANOVA test followed by Newman-Keuls test.

**Figure 5:**
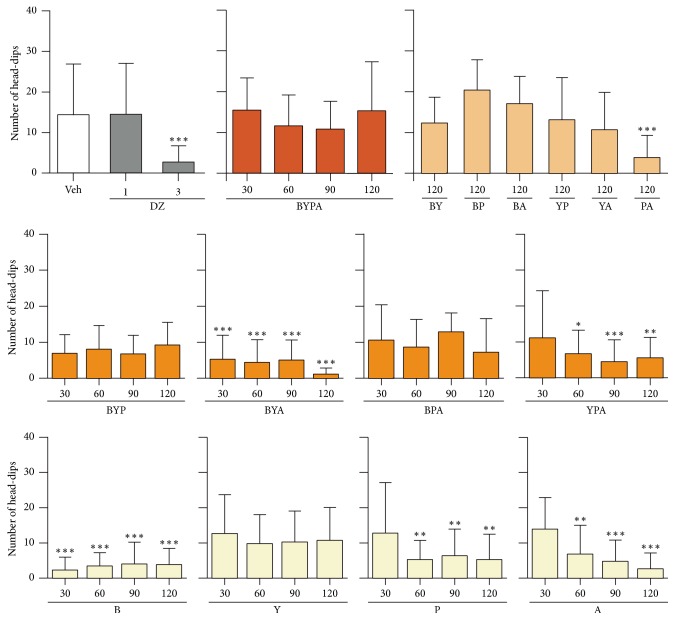
Sedative effects of different combinations of B, Y, P, and A herbs. The performances of mice treated with saline vehicle (Veh), diazepam (DZ), and different single-herb or multiherb extracts were assessed with respect to sedative effect using the hole-board test, where reduced head-dips represented a sedative effect. Data are expressed in mean ± SD of the number of head-dips. ^*∗*^(*p* < 0.05), ^*∗∗*^(*p* < 0.01), or ^*∗∗∗*^(*p* < 0.001) indicates significant difference from Veh group based on one-way ANOVA test followed by Newman-Keuls test.

The single-herb extracts of Y, P, and A but not that of B displayed anxiolytic effects as evidenced by significant increase in percentage of time spent in open arms in the elevated plus-maze test starting from an oral dose of 60 mg/kg. The B-, P-, and A-treated mice displayed sedative behavior as evidenced by significant decrease in head-dips in the hole-board test, starting from 60 mg/kg in the case of P- and A-treated mice, as well as starting from the even lower dose of 30 mg/kg in the case of B-treated mice. In contrast, the Y-treated mice did not display any sedation up to 120 mg/kg (Figures 4 and 5).

The two-herb BA and PA extracts induced significant anxiolytic effects, and only PA but not BA induced significant sedative effects. The three-herb extracts BYA and YPA yielded significant anxiolytic effect as well as sedative effect, whereas BPA gave rise to significant anxiolytic effect but no sedative effect up to 120 mg/kg. BYP did not give rise to any significant anxiolytic or sedative effect. As reported earlier [12], the four-herb BYPA combination induced significant anxiolysis but no sedative effect up to 120 mg/kg (Figures 4 and 5).

### 3.3. Locomotor Activity, Motor Coordination, and Anterograde Amnesia

**Figure 6:**
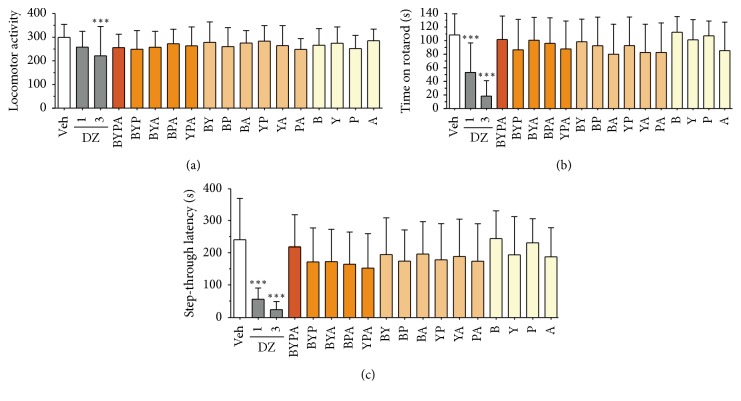
Absence of side effects in herbal extract-treated mice. (a) Locomotor activity evaluated using the locomotor activity test; (b) muscle coordination evaluated using the rotarod test; and (c) memory impairment evaluated using the step-through passive avoidance test. All herbal extracts were tested at the dose of 120 mg/kg, and DZ was tested at 1 mg/kg and 3 mg/kg. Data are expressed in mean ± SD. ^*∗∗∗*^(*p* < 0.001) indicates significant difference from Veh group based on one-way ANOVA test followed by Newman-Keuls test.

There was no significant change in any of the one-, two-, three-, or four-herb extract-treated mice at 120 mg/kg, whereas 3 mg/kg diazepam induced a significant reduction (*p* < 0.001), in locomotor activity (Figure 6(a)). There was also no significant change in the time mice managed to stay on the rotarod (Figure 6(b)) or in the step-through latency in the step-through passive avoidance test (Figure 6(c)) following administration of any of the herbal extracts tested at 120 mg/kg, whereas diazepam at either 1 mg/kg or 3 mg/kg reduced both the time on the rotarod and the length of step-through latency (*p* < 0.001). These findings indicated that none of the herbal extracts tested induced any significant side effect in terms of altered level of locomotor activity, reduced motor coordination on the rotarod, or anterograde amnesia in the step-through avoidance test.

### 3.4. Interactions with Neuroreceptors

**Figure 7:**
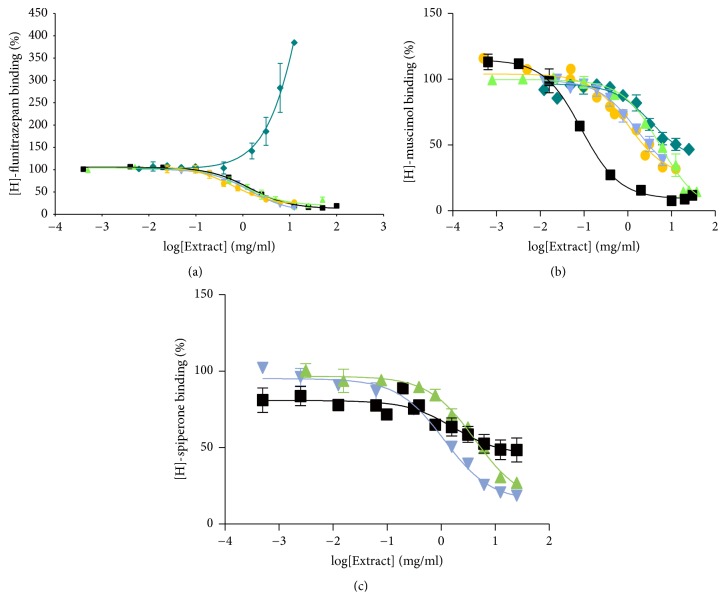
Effects on ligand binding to membrane neuroreceptors. Effects of extracts of BYPA (■, black); B (▲, green); Y (▼, light blue); P (♦, dark blue); and A (●, yellow). (a) Binding of [^3^H]-flunitrazepam yielding IC_50_ values of 1.37 ± 0.05 mg/ml, 1.05 ± 0.10 mg/ml, 1.41 ± 0.06 mg/ml, and 0.60 ± 0.12 mg/ml for BYPA, B, Y, and A, respectively, and EC_50_ of 4.01 ± 0.39 mg/ml for P. (b) Binding of [^3^H]-muscimol yielding IC_50_ values of 0.09 ± 0.06 mg/ml, 5.50 ± 0.08 mg/ml, 1.57 ± 0.12 mg/ml, 3.49 ± 0.14 mg/ml, and 1.02 ± 0.07 mg/ml, respectively, for BYPA, B, Y, P, and A and (c) binding of [^3^H]-spiperone yielding IC_50_ values of 1.71 ± 0.25 mg/ml, 4.26 ± 0.09 mg/ml, and 1.14 ± 0.07 mg/ml for BYPA, B, and Y, with little inhibition by P or A. Data represent mean ± SEM. Total [^3^H]-ligand binding recorded in each of panels (a)–(c) represented the sum of specific and nonspecific bindings (see Section 2.13), with specific binding constituting more than 90%, 75%, and 60% of total binding, respectively.

**Figure 8:**
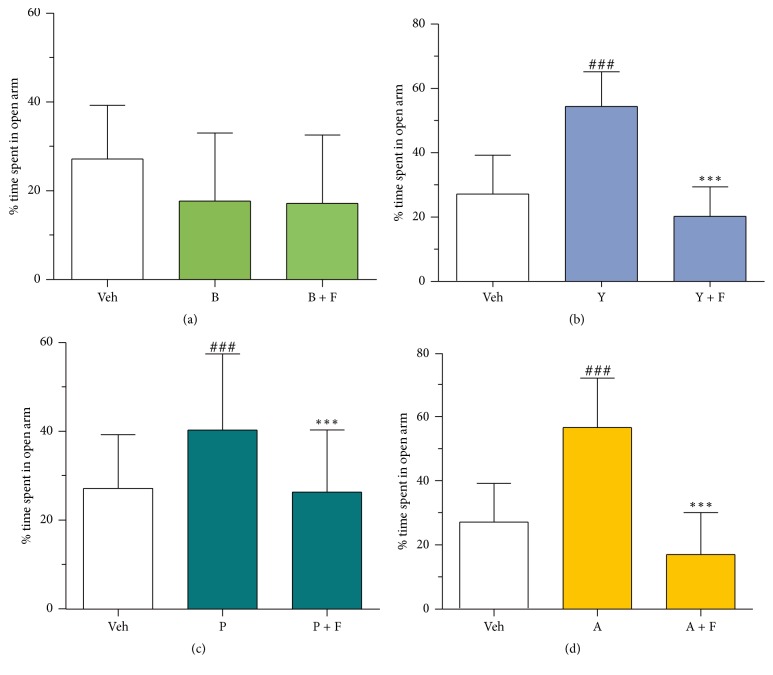
Anxiolytic effects of single herbs blocked by the GABA_A_ receptor BZ-site antagonist flumazenil. Mice were treated with saline vehicle (Veh), single-herb extract (120 mg/kg of oral B, Y, P, or A) with or without flumazenil (namely F, 1.25 mg/kg i.p). Anxiolytic effect was evaluated based on time spent in open arms. Data represent mean ± SD. ^###^(*p* < 0.001) indicates significant difference between test group receiving single herb without flumazenil and Veh group, whereas ^*∗∗∗*^(*p* < 0.001) indicates significant difference between test group receiving single herb with flumazenil and test group receiving single herb without flumazenil, based on one-way ANOVA test followed by Newman-Keuls test.

The B, Y, A, and BYPA extracts all inhibited the binding of the GABA_A_ receptor benzodiazepine- (BZ-) site agonist [^3^H]-flunitrazepam to rat cerebral cortex membranes with half-inhibition concentration (IC_50_) values within the same order of magnitude. However, cooperative binding was observed between P-extract and [^3^H]-flunitrazepam (Figure 7(a)). While the competitive binding of ingredients in Y, A, or BYPA to the BZ-site was consistent with the BZ-site playing a role in the anxiolytic effects of Y, A, and BYPA (Figure 4), the competitive binding of B ingredients to the BZ-site but lack of anxiolysis by B up to 120 mg/kg, as well as the cooperative binding of P to the BZ-site, was suggestive of complex interactions of the ingredients of B or P with GABA_A_ receptors. On the other hand, the sensitivity of Y-, P-, and A-induced anxiolysis, measured based on increase in time spent in open arms in the elevated plus-maze, to inhibition by the BZ-site antagonist flumazenil (Figure 8) was consistent with participation of the BZ-site in anxiolysis by Y, P, and A. In Figure 7(b), extracts of the single herbs B, Y, P, A, and the four-herb BYPA all inhibited the binding of the GABA_A_ receptor GABA-site ligand [^3^H]-muscimol to the membranes. However, the IC_50_ value of 0.09 mg/ml for BYPA was more than an order of magnitude lower than the IC_50_ values of 5.50 mg/ml, 1.57 mg/ml, 3.49 mg/ml, and 1.02 mg/ml for B, Y, P, and A, respectively, clearly pointing to the presence of synergism between the constituents of B, Y, P, and A with respect to [^3^H]-muscimol binding to the GABA-site. Extracts B, Y, and BYPA also inhibited membrane binding of the dopamine D2 receptor ligand [^3^H]-spiperone (Figure 7(c)), further demonstrating the breadth of interactions of the B, Y, P, and A ingredients with neuroreceptors that could contribute to the synergisms and antagonisms between these four medicinal herbs.

## 4. Discussion

Based on the d-galactose-induced accelerated-aging model, the present study showed that extracts of various mixtures of the B, Y, P, and A herbs, especially the BYP, BYA, BY, BP, YP, and BYPA extracts, brought about significant protection against one or more aging effects, including improvement of spatial memory (Figure 1), reduction in neuroinflammation in terms of the brain levels of proinflammatory cytokines TNF-*α* and IL-6, or reduction in oxidative stress in the brain in terms of the level of MDA (Figure 2). Notably, spatial memory deficit and enhanced neuroinflammation are encountered not only in normal aging but also in Alzheimer's disease (AD) [45–48], and increased oxidative stress in the brain is common to normal aging, AD, and Parkinson's disease (PD) [3, 4, 49–51]. In this regard, it has been suggested that combination therapy consisting of a drug targeting the amyloid-beta (A*β*) and/or tau protein together with a medication modulating neuroinflammation may delay the progression of AD [52]. Accordingly, the present findings suggest that the BYP, BYA, BY, BP, YP, and BYPA extracts represent potentially useful agents for the prevention and/or treatment of the effects of normal aging, AD, and PD. These extracts, as in the case of all other extracts comprising two or more of the B, Y, P, and A herbs, were devoid of significant side effects with respect to altered locomotor activity, decreased motor coordination on the rotarod, and anterograde amnesia in the passive step-through avoidance test at 120 mg/kg (Figure 6). The BYPA, BYP, BYA, BPA, and YPA extracts also have been found to protect facial tissues against the loss of whiskers (Figure 3). In addition, the BA, BPA, and BYPA extracts can provide anxiolysis, and the PA, BYA, and YPA extracts can provide both anxiolysis and sedation for the treatment of sleep disorders (Figures 4 and 5). All the extracts derived from B, Y, P, and/or A showed little evidence of adverse side effects regarding altered level of locomotor activity, reduced motor coordination, or anterograde amnesia (Figure 6).

**Figure 9:**
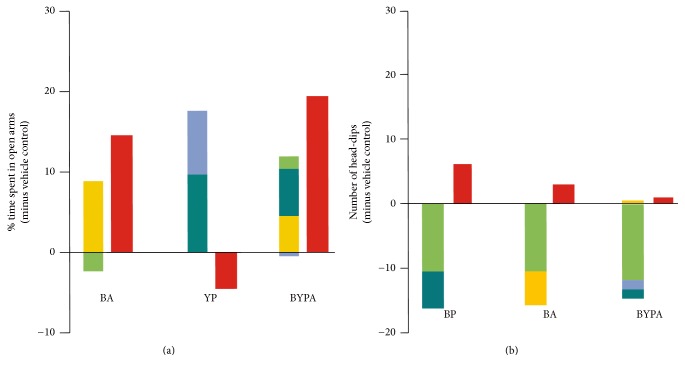
Synergisms and antagonisms between herbs. (a) Examples of synergism or antagonism with respect to anxiolytic effects measured based on time spent in open arms in elevated plus-maze test. The paired columns for herbal combinations BA and BYPA show in each instance that the anxiolytic effect induced by the combination (red column on the right) exceeded the sum of effects induced by the individual component herbs (composite column on the left: B in green, Y in light blue, P in dark blue, and A in yellow); in contrast, the paired columns for YP show that the anxiolytic effects induced by its component herbs Y and P were abolished by antagonisms within the YP combination. (b) Examples of antagonism with respect to sedative effects measured based on number of head-dips in the hole-board test. The paired columns for herbal combinations BP, BA, and BYPA show that the sedative effects induced by the component herbs in each instance (composite column on the left) were completely abolished by antagonisms within each combination. Sedative effects were estimated based on head-dips in the hole-board test. The anxiolytic or sedative effects represented by the combination columns were those indicated in Figures 4 and 5 for a dosage of 120 mg/kg. The individual effects of single herbs represented in the composite column were derived from the dose-response relationships for the single-herb data in Figures 4 and 5: the total dosage in each composite column was 120 mg/kg, and the contributions of individual herbs were estimated based on their weight ratios in the combination and interpolations of their individual dose-response curves.

Multiherb formulas of Chinese medicine are typically combination therapies that comprise complementary “Jun” (Sovereign), “Chen” (Minister), “Zuo” (Assistant), and “Shi” (Courier) herbs with the aim of multitargeting and optimizing therapeutic efficacy and reducing adverse side effects through mutual antagonisms and synergisms between the component herbs [8–11]. In the present study, examples of pronounced synergisms and antagonisms with respect to anxiolysis and antagonisms with respect to sedative effects were observed among component herbs (Figures 9(a) and 9(b)). The BZ-site of GABA_A_ receptors has been implicated in both anxiolysis and sedation [53, 54]. Furthermore, evidence indicated that aging is accompanied by reduced glutamic acid decarboxylase activity and GABA synthesis in hippocampal interneurons, resulting in enhanced neuronal excitability contributing to memory impairment [55–57], and GABA_A_ receptor modulators have been employed to improve both spatial memory and working memory [58, 59]. TNF-*α* could increase mitochondrial ROS production [60, 61], and ROS also could trigger proinflammatory cytokine expression* via* activation of NF-*κ*B [62]. Therefore the parameters of anxiolysis, sedation, spatial memory performance, brain MDA, TNF-*α*, and IL-6 levels could be intercorrelated, although the exact relationships among them yet await delineation.

Importantly, although each of the B, P, and A herbs when administered alone at 30 mg/kg or 60/mg upwards gave rise to significant sedation, none of the BYP, BPA, BY, BP, BA, YP, YA, and BYPA combinations induced significant sedation at 120 mg/kg (Figure 5). Since sedative drugs are difficult to administer over prolonged periods, the nonsedative property of BYP, BY, BP, YP, and BYPA on account of antagonisms between their component herbs usefully facilitates their chronic application as potential antiaging or antineurodegeneration agents.

## 5. Conclusions

In conclusion, oral administrations of various two-, three-, and four-herb combinations of Radix* Bupleurum chinense* DC (“B”), Rhizoma* Corydalis yanhusuo* WT Wang (“Y”), Caulis* Polygonum multiflorum* Thunb (“P”), and Flos* Albizia julibrissin* Durazz (“A”) have been found to alleviate spatial memory deficit and decrease the brain levels of TNF-*α*, IL-6 and MDA, and/or loss of whiskers in the d-galactose-induced accelerated-aging mouse model. In addition, some of the combinations induced anxiolytic effects unaccompanied by sedative effects, sedative effects unaccompanied by anxiolytic effects, or both anxiolytic and sedative effects, all without any significant side effects with respect to locomotor activity, motor coordination, or anterograde amnesia. These results therefore suggest that the two-, three-, and four-herb combinations of these four medicinal herbs could provide potentially useful oral-active therapeutic agents or nutraceuticals for the prevention and/or treatment of normal aging, Alzheimer's disease, Parkinson's disease, anxiety disorders, and/or sleep disorders. The findings also suggest that the ingredients of these four medicinal herbs furnish a promising source for the isolation and identification of single-compound agents useful for the prevention and/or treatment and usage as experimental probes into the etiology and pathophysiology of clinical conditions important to normal aging or age-related neurodegeneration. Furthermore, since various combinations of B, Y, P, and A could protect against accelerated aging both in the brain and in facial tissues, comprehensive investigations are called for to determine the scope and magnitude of the antiaging effects exerted by varied combinations of these four herbs on the changes in different human tissues and organs in the course of normal and pathological aging.

## Figures and Tables

**Table 1:** Prevention of loss of whiskers in D-galactose-induced aging mice.

Treatment group	Loss of whiskers (% mice)	
	0	+
Veh	100	0
d-gal^*∗*^	50	50
d-gal + BYPA	100	0
d-gal + BYP	100	0
d-gal + BYA	100	0
d-gal + BPA	100	0
d-gal + YPA	100	0

*n* = 12 mice/group. “0”: no loss of whiskers; “+”: loss of whiskers.

^*∗*^Based on two-sided Fisher's exact test, the d-gal group with 6/12 whisker loss differed significantly from the Veh group with 0/12 whisker loss (*p* = 0.014), from each of the BYPA, BYP, BYA, BPA, and YPA extract-treated groups showing 0/12 whisker loss (*p* = 0.014), and from a combined herbal-treated group with 0/60 whisker loss (*p* = 5.9 × 10^−6^). Photographs of representative mice from the different treatment groups are illustrated in Figure 3.
